# Shear stress affects the architecture and cohesion of *Chlorella vulgaris* biofilms

**DOI:** 10.1038/s41598-021-83523-3

**Published:** 2021-02-17

**Authors:** A. Fanesi, M. Lavayssière, C. Breton, O. Bernard, R. Briandet, F. Lopes

**Affiliations:** 1 Laboratoire Génie des Procédés et Matériaux (LGPM), CentraleSupélec, Université Paris-Saclay, 91190 Gif-sur-Yvette, France; 2 Biocore, INRIA, Université Côte d’Azur, 06902 Sophia Antipolis Cedex, France; 3 Université Paris-Saclay, INRAE, AgroParisTech, Micalis Institute, 78350 Jouy-en-Josas, France

**Keywords:** Biofilms, Plant physiology, Confocal microscopy

## Abstract

The architecture of microalgae biofilms has been poorly investigated, in particular with respect to shear stress, which is a crucial factor in biofilm-based reactor design and operation. To investigate how microalgae biofilms respond to different hydrodynamic regimes, the architecture and cohesion of *Chlorella vulgaris* biofilms were studied in flow-cells at three shear stress: 1.0, 6.5 and 11.0 mPa. Biofilm physical properties and architecture dynamics were monitored using a set of microscopic techniques such as, fluorescence recovery after photobleaching (FRAP) and particle tracking. At low shear, biofilms cohesion was heterogeneous resulting in a strong basal (close to the substrate) layer and in more loose superficial ones. Higher shear (11.0 mPa) significantly increased the cohesion of the biofilms allowing them to grow thicker and to produce more biomass, likely due to a biological response to resist the shear stress. Interestingly, an acclimation strategy seemed also to occur which allowed the biofilms to preserve their growth rate at the different hydrodynamic regimes. Our results are in accordance with those previously reported for bacteria biofilms, revealing some general physical/mechanical rules that govern microalgae life on substrates. These results may bring new insights about how to improve productivity and stability of microalgae biofilm-based systems.

## Introduction

In recent years, biofilm-based reactors have been proposed as a valuable alternative to traditional photo-bioreactors for microalgae cultivation due to a series of advantages such as an easier biomass harvesting procedure, lower operative costs and higher productivities^1–3^.

Several microalgae strains have been tested under many growth conditions revealing that such systems are able to support high productivities^4^. However, although one of the main characteristics affecting biofilm activity and properties is their architecture (i.e. peculiar spatial organization of cells and matrix components such as exopolysaccharides), only few works investigated how the architecture of microalgae biofilms changes as a function of the strain and growth conditions^5–10^. Recently, Fanesi et al.^9^ reported that microalgae biofilms architecture are species-specific. These results have important consequences for optimal process design to maximize productivity.

Besides photon flux density, the hydrodynamic regime in a reactor is one of the key factors for the development of microalgae biofilms^4^. Indeed, reactor geometries may generate different flow and shear patterns that in turn induce the formation of specific flow-related biofilm structures^11^. For bacterial biofilms, the effect of shear stress on their development and architecture and physical properties has been previously characterized: at low shear the biofilms appear loose and thick whereas at high shear they become denser and thinner^12–14^. A high shear also induces the development of biofilms with a higher cohesion, and erosion tests have demonstrated the presence of a very resistant basal (close to the substrate) biofilm layer^15,16^. Overall, these structural shifts have been proposed to be a strategy aimed at optimizing the inner nutrient transport and persistence on the substrate. These interpretations were supported by mathematical simulations provided by several models tracking biofilm architecture, mechanical strength and detachment which predicted biofilm growth and organization in several applied contexts^17–19^. It is clear that the hydrodynamic regime used for biofilm cultivation can impact in parallel their productivity and stability rising non-obvious challenges for process control^12–14^. For instance, thick biofilms, frequently developed under low shear stress, may become limited due to a low transport of nutrients^20^. On the other hand, mass transport and growth will be improved in conditions of higher flow rate/rotation speed, but this will lead to higher detachment thus affecting process stability^21^. Therefore, a good balance among these factors must be reached.

Only few studies investigated structures development in microalgae biofilms under flow^10,22^, and a detailed comprehension of how hydrodynamic conditions alter their architecture, physical and cohesion properties is still missing^23^. However, such information would be of paramount importance to deeply understand biofilm-based systems and to improve the design of such reactors^24^. Based on the information about bacterial biofilms and shear stress, we hypothesized that in a similar manner microalgae biofilms would undergo architectural and physiological transitions to ensure their persistence on the substrate. In order to test such hypothesis, the biofilms of *Chlorella vulgaris* were cultivated in flow-cells at several shear stress and their architecture characterized by confocal laser scanning microscopy (CLSM) and image analysis. Advanced microscopy techniques such as fluorescence recovery after photobleaching (FRAP) and particle tracking have been particularly useful in characterizing diffusion and the rheological properties of bacterial biofilms^25,26^ and were therefore here employed together with an erosion test to determine the physical and cohesion properties of microalgae biofilms as a function of the shear stress.

## Results

### Microalgae biofilms architecture at different hydrodynamic shears

**Figure 1:**
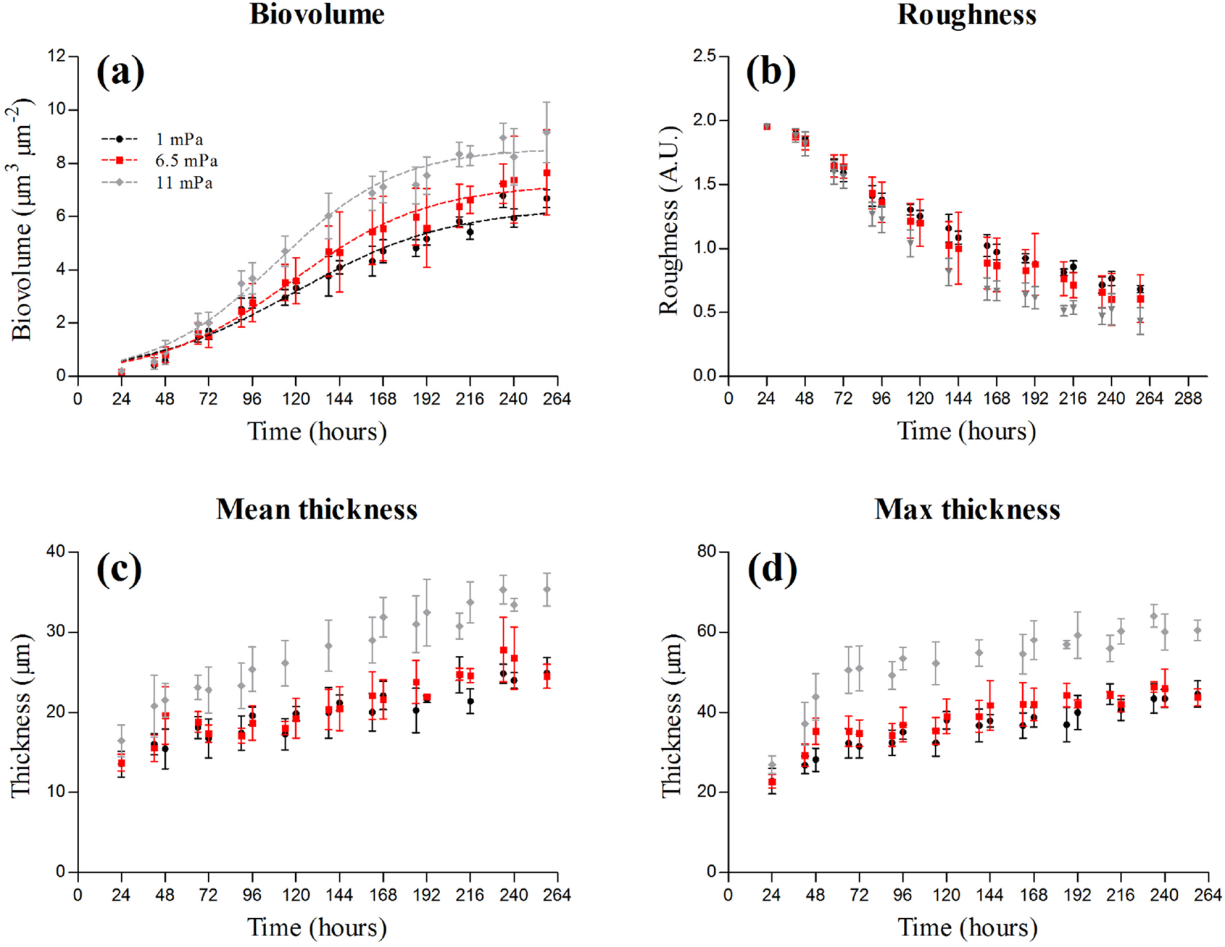
Structural parameters of *C. vulgaris* biofilms grown in flow-cells at three hydrodynamic shears (1.0, 6.5 and 11.0 mPa). Biovolume (**a**), roughness (**b**), mean (**c**) and max thickness (**d**) are reported as function of time. The biovolume curves were fitted using the logistic function (dashed lines). The results are reported as the mean and standard deviation of at least three independent biological replicates. Error bars represent the standard deviation (n ≥ 3).

Regardless of the hydrodynamic conditions, biofilm development resulted in an increase of biovolume and thickness (mean and max) and a parallel decrease in roughness over time (Fig. 1a–d). However, the hydrodynamic regime affected the extent to which these changes occurred. The biovolume reached at the *plateau* by *C. vulgaris* significantly increased as a function of the shear stress (Fig. 1a and Fig. S1b) and was the highest in the biofilms grown under the highest shear (8 µm^3^ · µm^−2^; p < 0.05; Fig. 1a and Fig. S2b). The same was true also for the thickness, at 11.0 mPa the biofilms were thicker than those grown at 1.0 and 6.5 mPa (Fig. 1c,d). After 72 h of development, the biofilm developed at 11 mPa presented a slightly lower roughness coefficient than the biofilms grown at 1 and 6.5 mPa (Fig. 1b).

### Growth and physical properties of microalgae biofilms under flow

The growth rate of the biofilms did not change as a function of the shear and was on average 0.8 d^−1^ (p > 0.05; Fig. S1a).

**Figure 2:**
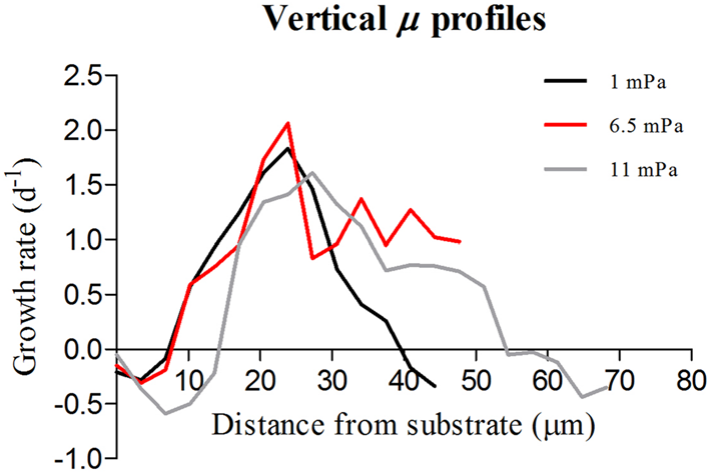
Vertical profile of growth rate in the biofilms formed by *C. vulgaris* grown at three hydrodynamic shears: 1.0, 6.5 and 11.0 mPa. The growth rate for each layer was calculated as the slope of the linear regression between the coverage of cells in each layer over time. The data are reported as the mean of at least three independent biological replicates (n ≥ 3).

The profile of growth rate over depth revealed interesting results concerning the optimal spatial localization of cell growth in the biofilms (Fig. 2). At all conditions, the deeper layers of the biofilm (z = 0–15 µm) did not present any growth. Between 15 and 20 µm from the substrate the *µ* of the layers increased and presented maximal values between 20 and 30 µm. The layers positioned at greater distances presented on the other hand a decreasing trend of *µ*.

Typical FRAP analysis are reported in Fig. S2. FRAP curves were very similar at all the shears (Fig.S2b). The diffusion coefficient of 70 kDa FITC-dextran in the biofilms obtained from the FRAP curves was on average ~ 45 µm^2^ · s^−1^ and did not change significantly as a function of the shear (p > 0.05; Fig. S2c). Furthermore, the diffusion coefficient in all biofilms was similar to that obtained in the growth medium (Figure S2c). Also, at all conditions the fluorescence was able to recover to its full intensity after photobleaching (i.e. 1 A.U.; Fig. S2b), suggesting the absence of immobile fraction of the diffusing fluorescent probes (Fig. S2b). To the best of our knowledge, this is the first report about diffusion coefficients in photosynthetic biofilms.

The particle tracking analysis revealed that the cells were tightly attached to the substrate at all conditions (0.6–1 µm of displacement; Fig. S3–S4). Regardless from the hydrodynamic regime, the shape of the probability functions did not change remarkably over time. However, the modes of the density functions were centred at different displacements values at day 2 and 4. In particular, at day 4 the cells in the biofilms developed at 1.0 mPa presented the greater displacement (Fig. S3–S4).

**Figure 3:**
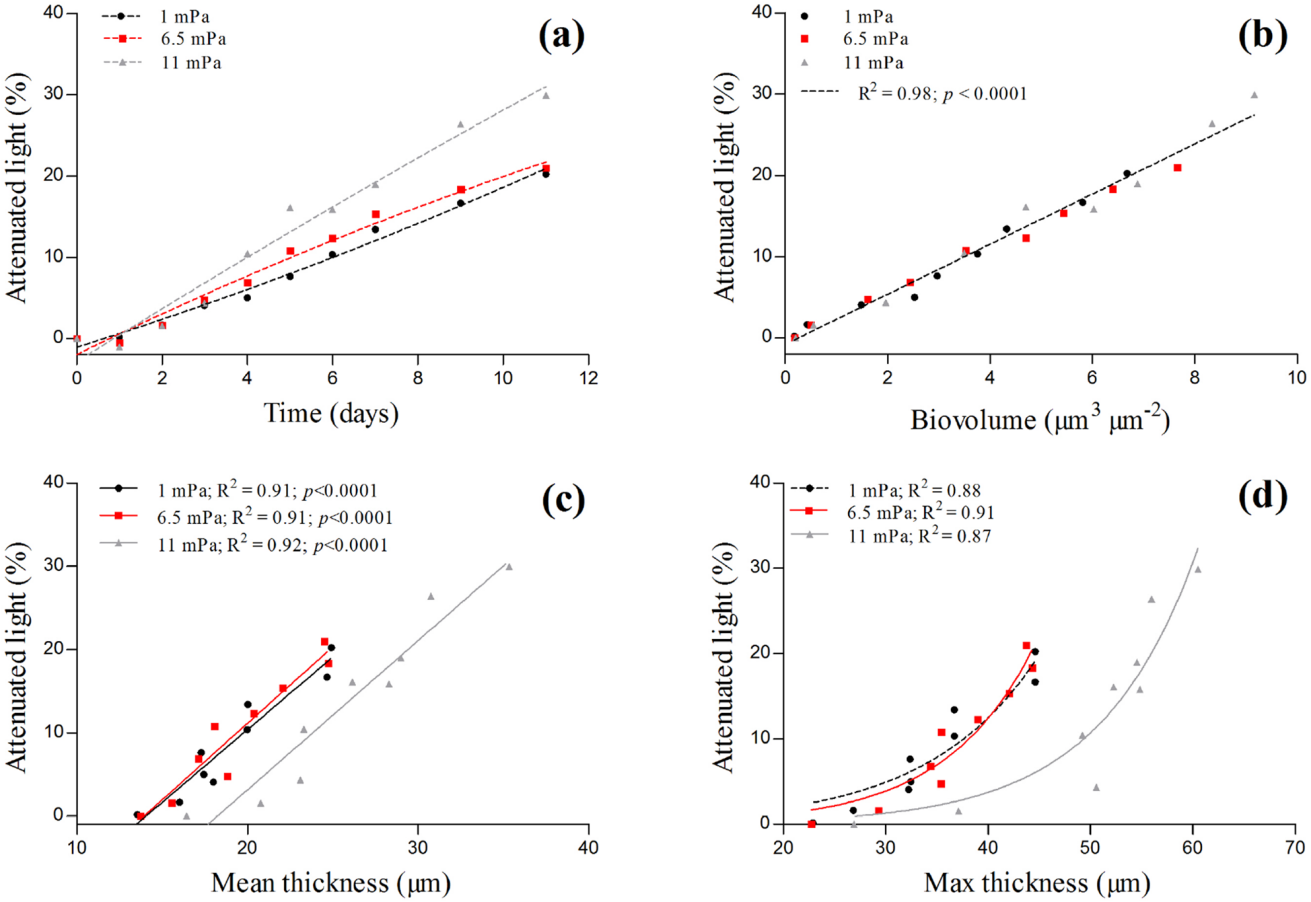
Attenuated light in the biofilms of *C. vulgaris* grown at three different hydrodynamic shears (1.0, 6.5 and 11.0 mPa). In (**a**) the attenuated light is shown as a function of time. The relationships among the light attenuated in the biofilms and the structural parameters biovolume (**b**), mean thickness (**c**) and maximal thickness (**d**) of the biofilms are also reported. The data represent the mean of at least three independent biological replicates (n ≥ 3).

The attenuation of light by the biofilms increased over time regardless of the shear stress. At the end of the experiments, the biofilms attenuated 20–30% of the incident light (Fig. 3a). A positive linear relationship was found between the light attenuated by the biofilms and their biovolume (R^2^ = 0.98; p < 0.0001; Fig. 3b). Therefore, the higher biovolume developed at 11.0 mPa was also reflected in the higher percentage of light attenuated by the biofilms at the end of the experiments (30%). Interestingly, when considering the light attenuated by the biofilms and their mean and maximal thickness two different relationships were found (Fig. 3c,d). The light attenuated by the biofilms was linearly correlated to their mean thickness (Fig. 3c), but the relationships deviated from linearity and resembled to an exponential function when considering the maximal thickness of the biofilms (Fig. 3d). Furthermore, two separated relationships where found for the biofilms grown at 1.0 mPa and 6.5 mPa and those grown at 11.0 mPa. At a determinate mean or maximal thickness, the biofilms grown at 11.0 mPa attenuated less light, with respect to those grown at 1.0 and 6.5 mPa (Fig. 3c,d).

### Cohesion of microalgae biofilms

**Figure 4:**
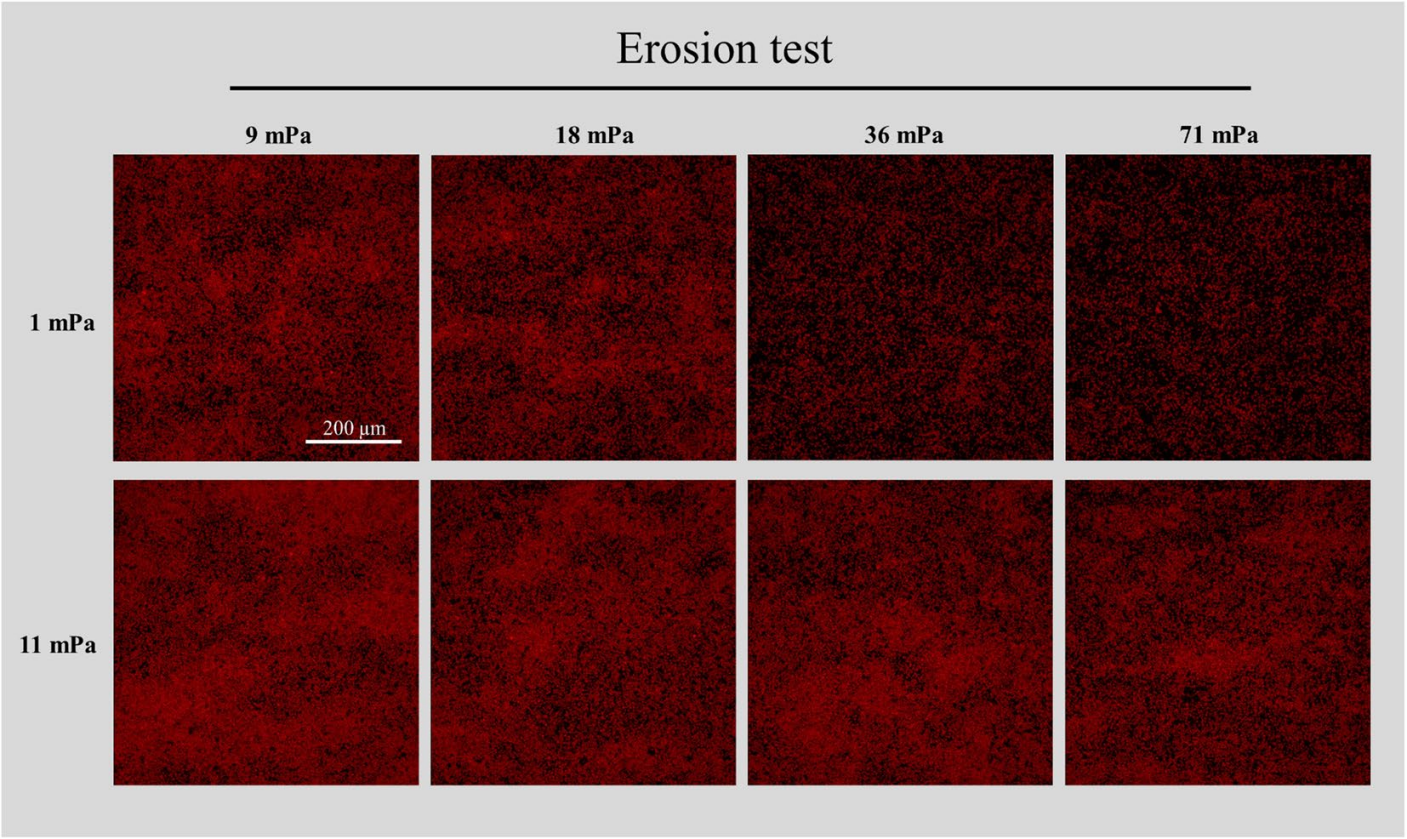
Max intensity projections of representative CLSM stacks acquired during the erosion test. The biofilms grown at three shears (1, 6.5 and 11.0 mPa) were subjected to four (9, 18, 36 and 71 mPa) increasing shears (10 min for each shear) and the biovolume of the biofilms still present in the flow cell was measured after each step by CLSM in order to test the cohesiveness of *C. vulgaris* biofilms. Images are shown for the biofilms grown at 1 and 11.0 mPa and the brightness has been adjusted for visual purposes.

**Figure 5:**
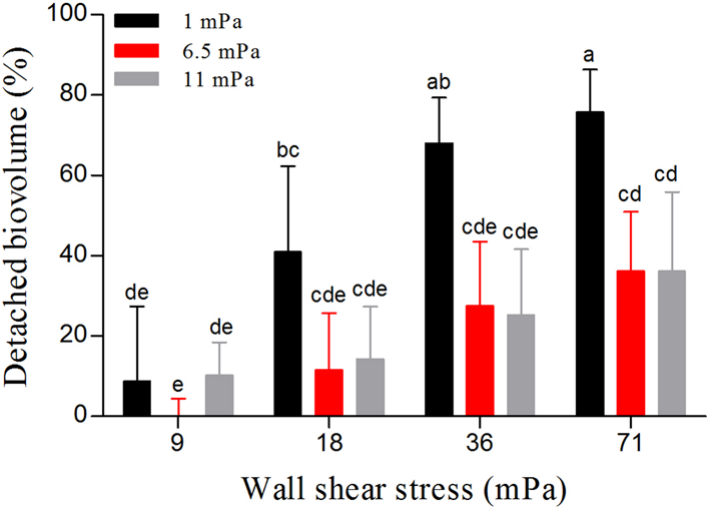
Erosion test performed on the biofilms grown at three different hydrodynamic regimes (1.0, 6.5 and 11.0 mPa). The biofilms were subjected to an increasing set of shears (9.0, 18, 36, and 71 mPa), each step lasted 10 min. The biovolume detached at each shear was calculated with respect to the initial biovolume (pre-erosion test). The results are the mean and standard deviation of at least three independent biological replicates (n ≥ 3). Bars with different letters represent statistically different means (*p* < 0.05) as determined by pair-wise comparisons after two-way ANOVA considering both the growth shear stress and the shear stress applied during the erosion stress as factors.

The biovolume detached from the biofilms grown at 1.0 mPa was higher than that lost from the biofilms grown at 6.5 and 11.0 mPa (p < 0.05; Fig. 4 and Fig. 5). On the other hand, no statistically significant difference was found between 6.5 and 11.0 mPa (p > 0.05; Fig. 5). At the end of the erosion test, the biofilms grown at the lowest shear (1.0 mPa) lost more than 70% of their biovolume (Fig. 5). On the other hand, the biofilms grown at the higher shear stress presented only 35% of biovolume detached (Figs. 4 and 5).

**Figure 6:**
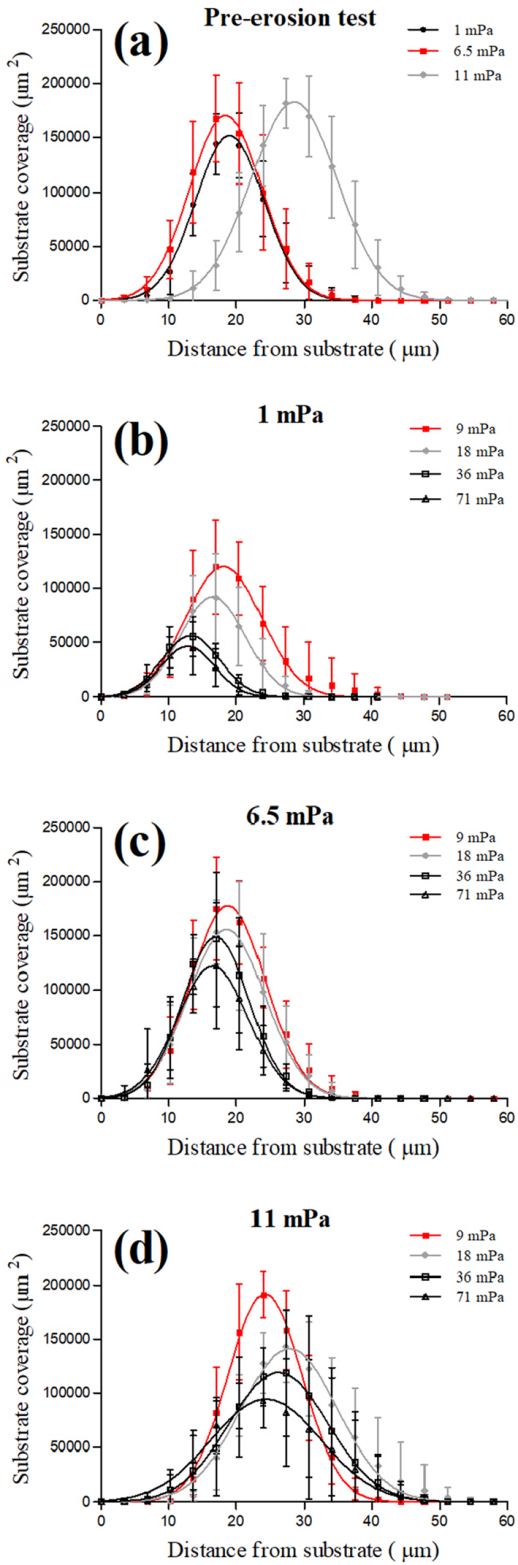
Vertical profiles of cell coverage in the biofilms during the erosion test. Panel (**a**) shows the vertical profile before the erosion test. Panels (**b**–**d**) show the vertical distribution of cells after the application of the erosion test for the biofilm grown at three hydrodynamic shears: 1.0, 6.5 and 11.0 mPa, respectively. The biofilms were subjected to an increasing set of shears (9.0, 18, 36, and 71 mPa), each step lasted for 10 min. Error bars represent the standard deviation (n ≥ 3).

When considering the vertical profile of cell coverage obtained during the erosion test, the loss of biovolume in the biofilms grown at low shear (1 mPa) was located at the most superficial layers (Fig. 6a,b). At the end of the erosion test, only the deepest biofilm layers remained attached to the substrate and most of the cells detached decreasing the coverage of the substrate from 120,000 to 45,000 µm^2^ (Figs. 4 and 6b). For the two other tested conditions (6.5 and 11.0 mPa), although biofilm detachment seemed to be mainly related to the upper layers, no significant change in vertical profile of cell coverage was observed with increased applied shear stress (Fig. 6a,c,d).

## Discussion

The aim of this study was to give more insight on the effect of hydrodynamics on microalgae biofilm growth, which has been so far poorly studied^10,22,23^. Our results confirm the hypothesis that microalgae biofilms respond to different hydrodynamic shears rearranging their architecture. Moreover, this reorganization seems to occur at multiple levels of biofilm architecture: from their mechanical properties to their 3D structure.

In accordance with the literature on bacteria, the biofilms of *C. vulgaris* developed at different shear stress appear to modulate their cohesiveness^13,16,27^. The biofilms grown at the lowest shear stress (1.0 mPa) were loose and after an erosion test, only a basal layer remained on the substrate (Figs. 4 and 6). On the other hand, the biofilms grown at 6.5 and 11.0 mPa remained mostly unperturbed (Figs. 4, 5 and 6). The mechanical strength of the basal layer was further revealed by the particle tracking analysis that confirmed the presence of firmly attached cells on the substrate, regardless of the shear conditions (Figs. S3 and S4). Although several studies about bacterial biofilms described a vertical heterogeneity of cohesion and the presence of a compact basal layer resistant to detachment^15,16,18,27^, to the best of our knowledge this is the first report for microalgae biofilms. The consistency of our results with those reported on bacterial biofilms have a great valence for the general interpretation of 3D structure of biofilms revealing intrinsic general rules that govern life on surfaces no matter the microorganism considered.

The greater cohesion of bacterial biofilms developed at high shears has been attributed to an higher density of the biofilms and/or different contents of matrix exopolysaccharides^15,16,18,27–30^. It is likely that extracellular macromolecules, such as exopolysaccharides, proteins, eDNA and lipids played a crucial role in the cohesion of the biofilms altering the qualitative properties of the matrix^31^. Furthermore, local hot-spots of matrix components^28^, or different cell–cell or cell–matrix interaction might have as well modified the cohesiveness of the biofilms^32,33^. A more in-depth characterization of matrix components in monospecific microalgae biofilms grown under flow should therefore be performed in further works.

The greater cohesiveness of the biofilms grown at higher shear appeared to have important repercussions on their architectural dynamics (Fig. 1). At 11 mPa, *C. vulgaris* developed biofilms with a greater biomass and thickness, reflecting a lower detachment (Fig. 1a,c,d). Similar results have been reported also for marine microalgae grown in flow-cells at two flow rates^10^. Although such architectural rearrangements may affect nutrient transport^13^ and light availability^23^, the biofilm as an assembly has coping mechanisms to ensure the persistence of the 3D structure on the substrate. For instance, cell-induced electrochemical signals in biofilms of *Bacillus subtilis* allow to avoid nutrient starvation in the interior of the colonies as their size reaches a threshold value^34^. In a similar way, phototrophic organisms can alter their pigments content over the depth of a biofilm to ensure light penetration as the community develop^35^. These mechanisms might be seen as acclimation responses initiated to promote simultaneously biofilm activity and persistence in a determinate environment. In line with these experimental observations, *C. vulgaris* biofilms presented identical growth rates at the different shears despite their architectural differences (Fig. S1a). This suggests that even though their 3D structures were different, nutrients and light were reaching the cells in a similar manner no matter the hydrodynamic conditions tested. On the base of our results, we concluded that this was linked to an unchanged diffusion coefficient in the biofilms (as determined using 70 kDa dextran as a molecular probe; Fig. S2) and to different optical properties of those biofilms (Fig. 3c,d). Such adjustments were likely the outcome of an acclimation strategy at the whole biofilm scale that allowed to maintain a constant growth rate even at a higher shear stress (Fig. 1a and Fig. S1a). This explanation is further supported by the similarity of the vertical growth rate profiles at all the hydrodynamic regimes (Fig. 2).

Spatial variations of growth rate and metabolic stratification have been described in bacterial biofilms^36^ where specific functions localize at precise depths of a biofilm as consequence of abiotic gradients^37,38^. In this study for the first time, we quantified the spatial variations of growth rate in microalgae biofilms. Biofilms are strongly stratified, and an optimal niche may result from the balance between several factors^36^. For instance, in the layers where nutrients and light are not limiting and shear is not so harsh, cells may divide and remain in the biofilm. Regardless of the shear, the layers comprised between 20 and 40 µm above the substrate exhibited the higher growth rate (up to 2 d^−1^). This zone may be interpreted as an optimal niche for cell growth within the biofilm core. This is also in agreement with what was postulated by Schnurr et al.^39^, which proposed that the most active layers are present in the superficial portion of phototrophic biofilms. Besides, the different relationships (linear and exponential) between the attenuated light by the biofilms and their mean and max thickness suggest heterogeneity of cell density and structure within the biofilms^9^ which impacted light attenuation. To the best of our knowledge, only few studies reported about the relationship between light attenuation and structural parameters in microalgae biofilms^39,40^.

In conclusion*,* by coupling the use of flow-cells for biofilm growth to a set of microscopic techniques we were able to demonstrate that shear stress plays a crucial role in microalgae biofilm development. At low shears, microalgae biofilms present a lower stability and a vertical gradient of cohesion strength resulting in loose top layers. Nevertheless, even after the application of a higher shear stress a basal biofilm layer is always persisting on the substratum making it possible for the biofilm to pursue the production of biomass. Under high shears, the biofilms have a higher cohesion that results in thicker biofilms with more biomass but unaltered growth rate, likely thanks to an acclimation strategy that allows the light and nutrients to penetrate deep in the biofilms. These results demonstrate how under controlled laboratory conditions the change of only one culture parameter lead to a cascade of effects with important consequences for the production of biomass and the mechanical stability of *C. vulgaris* biofilms. The control of biofilm systems for an optimal and maximal operation in outdoor conditions will require further experimental knowledge about microalgae biofilm organization (i.e. architecture, biochemical composition and metabolic activity) and the development of powerful predictive models for a realistic comprehension of such complex systems.

## Materials and methods

### Planktonic culture maintenance, cell counts and light measurements

*Chlorella vulgaris* SAG 211-12 (Göttingen, Germany) was cultivated in 3 N-Bristol^41^ in 250 mL Erlenmeyer flask filled with 60 mL of growth medium. The cultures were kept in the exponential phase (maximum cell density of about 3 × 10^6^ cell mL^−1^) by frequent dilutions (every 3 days). The flasks were continuously shaken in an incubator under a photon flux density of 20 µmol photons m^−2^ s^−1^ at 25 °C.

Cell counts were performed using a Guava easyCyte 5 flow cytometer (Millipore corporation 25,801 Industrial Blvd Hayward, CA94545). Chlorophyll *a* was excited at 488 nm and fluorescence was detected at 680 nm.

### Biofilms cultivation in flow-cells: inoculum, initial adhesion and growth

For the cultivation of biofilms under hydrodynamic conditions a flow through system consisting of a medium bottle, a peristaltic pump 205S/CA (Watson and Marlow; La Queue Lez Yvelines, France), bubble traps, milli-fluidic flow-cells and a trash bottle was used as previously described^10,42,43^. All the parts of the system were connected using silicon tubes with an internal diameter of 1 mm and by polypropylene connectors (Ibidi GmbH, Germany). Biofilms were grown in commercially available 3-channel flow-cells (ACCFL0001, IBI Scientific, Iowa, US) with dimensions 4 × 40 × 1 mm (*w, l* and *h*) where the growth substratum was represented by a glass coverslip already mounted on the flow-cells.

Before injecting the microalgae in a flow-cell, a solution of 0.5% (v/v) of sodium hypochloride was flown in the whole set-up at room temperature (25 °C) for at least two hours to sterilize it. After that, the system was emptied and fully rinsed with two litres of sterilized distilled water. Growth medium was then pumped inside the system.

The inoculum was prepared by diluting a volume of planktonic culture to a final concentration of 2 × 10^6^ cell · mL^−1^ and by injecting 1 mL of such suspension in the flow-cell through an in-line luer injection port (Ibidi GmbH, Germany). This cell concentration corresponded to a starting biovolume of sessile microalgae of ~ 0.2 µm^3^ · µm^−2^; see next section). The flow-cells were then closed with clips at each side and the cells were left for 24 h adhering to the surface of the coverslip under a continuous photon flux density of 70 µmol photons m^−2^ s^−1^. After 24 h, the clips were opened and the medium was pumped using a peristaltic pump (Watson and Marlow 205S). The biofilms were grown for 11 days (a time interval typically sufficient to reach the *plateau* of the biofilm development) under a continuous photon flux density of 70 µmol photons m^−2^ s^−1^ and at 25 °C. To impose different hydrodynamic regimes biofilms were cultivated at three flow rates (50, 300 and 500 µL min^−1^) corresponding to three different hydrodynamic shears: 1.0, 6.5 and 11.0 mPa. In all cases, the flow was laminar (Reynolds number in the range 0.3 to 3.0). Shear stress (τ; in mPa) was calculated with the formula presented in Hart et al.^26^ as follow:

1 $$\tau = \frac{6Q\upmu }{{wh}^{2}}$$

where *Q* is the flow rate in mL/min, *µ* is the dynamic viscosity of water at 25 °C (0.89 mPa · s), *w* and *h* are the width and height of the flow cell channel respectively.

### Microalgae biofilms structural dynamics

The biofilms were scanned twice a day using a confocal laser scanning microscope (CLSM) to obtain z-stacks of cells signal.

The confocal microscope set-up was similar to that described in Fanesi et al.^9^. Briefly, the images were acquired using an inverted Zeiss LSM700 confocal microscope (Carl Zeiss microscopy GmbH, Jena, Germany) equipped with a LD Plan-Neofluar 20×/0.4 Korr M27 objective with a 0.4 N.A. (numerical aperture). Each image (512 × 512 pixels) was 638 · 638 µm in size with a z-step of 3.4 µm and a lateral resolution of 1.25 µm. Each flow-cell was scanned in four random positions, from the inlet to the outlet, along the length of each channel.

Microalgae cells were detected by chlorophyll *a* auto-fluorescence. Chlorophyll *a* was excited at 639 nm and was detected using a low pass filter 615 nm.

A custom script running in Fiji^44,45^ was used to extract from the z-stacks the quantitative parameters used to characterize biofilm architecture: biovolume, maximal and mean thickness, roughness and the area occupied by the cells in each layer of a biofilm. The structural parameters were calculated according to the computation described in COMSTAT 2.1 (Technical University of Denmark;^42^), but the thresholding was performed using the Otsu algorithm^46^ considering the histogram of the whole stack.

In order to quantitatively compare the biofilm growth curves, they were fitted using the logistic function as described in Wang et al.^47^. From the curve fit, growth parameters such as the max specific growth rate (*µ*) and the biovolume reached by a biofilm at the *plateau* were obtained.

In order to characterize the growth of the different layers of *C. vulgaris* biofilms, the growth rate of each biofilm layer was quantified by converting the cell coverage area in each layer to natural logarithm and plotting it against time. A linear regression was fitted to the linear part of the curves and the *µ* of each layer calculated as the slope of the regression. Typically, the regressions were performed on at least four time points. In this way, a profile of growth rate over depth in the biofilms could be obtained.

### Light attenuation

Photon flux density was measured using a light meter (LI-190/R; LI-COR Biosciences GmbH, Bad Houmburg, Germany). Light attenuation by the biofilms was monitored by placing the flow-cells on the sensor of the light meter. Light attenuation was measured in three positions along the length of each channel present in the flow-cells. The attenuation of light was expressed with respect to the transmitted light measured before injecting the cells in the flow-cells (at day 0) as follow:

2 $${light\, attenuation}_{\left(i\right)}(\%)=1-\frac{{transmitted\, light}_{(i)}}{{transmitted\, light}_{(0)}} \cdot 100$$

where *i* represents the day during the growth of the biofilms.

### Diffusion coefficients—Fluorescence recovery after photobleaching (FRAP)

FRAP measurements are typically used to determine the diffusion properties of fluorescent molecules in biological samples by following the recovery of fluorescence after the application of a strong laser pulse (Fig.S2a,b). This technique has been already applied to estimate diffusion coefficients in bacterial biofilms^25^, but to the best of our knowledge it has never been applied to microalgae biofilms.

FRAP measurements were performed in one of the channels of a flow-cell at day 11 and repeated in multiple positions of the biofilms (5–15 positions) in order to account for spatial heterogeneity. Measurements were performed at the deepest layer of the biofilms (i.e. the one close to the substrate). The FRAP protocol was similar to the one described in Waharte et al.^25^. A solution of FITC-dextran 70 kDa (Molecular probes, Eugene, Oregon, USA) in Bristol was prepared with a final concentration of 350 µg mL^−1^. An aliquot of 160 µL of such solution was injected in the flow-cell and incubated in the dark for 30 min. Dextran was excited at 488 nm and detected using a band pass filter (490–530 nm). An 8 × digital zoom and a pixel resolution of 0.23 µm were selected to visualize a squared area of 27 × 27 µm (118 × 118 pixels; 16-bit). Within this area, two circular regions of interest (ROIs; 13 µm of diameter), one that would be bleached, and one used as a control (to correct for bleaching effect), were selected. Under these settings, images were acquired every 0.18 s (pixel dwell of 1.91 µs) and the total recovery curve was measured in 55 s. The protocol started with the acquisition of 20 images at low laser power (0.6%; 2.5 digital gain) and then a sequence (typically 3–4) of pulses emitted at 100% of the laser power were used to bleach the ROI until the fluorescence signal dropped to 40% of the initial intensity. At the same time, the dynamic of fluorescence was also monitored in the control ROIs (i.e. the not bleached area).

The recovery curves were subjected to a double normalization with respect to the control ROI and to the pre-bleach fluorescence. In this way the intensity ranged between 0 and 1 and was corrected for possible photo-fading effects over time. In order to obtain the diffusion coefficient of dextran in the biofilm, the curves were finally fitted using the model of Soumpasis^48^ (*D* = *r*^2^*/4τ*_*d*_; *r* is the radius of the bleached ROI and *τ*_*d*_ is the diffusion time obtained from the fitting). Measurements were also performed in flow-cells filled with Bristol medium (and no algae) in order to obtain the diffusion coefficient of FITC-dextran in the growth medium as a control. All recovery curves were measured at 25 °C. The mean fluorescence intensity of the ROIs was obtained in FIJI^44^ using the plug-in Time series analyser V3 (available online: https://imagej.nih.gov/ij/plugins/time-series.html) for time series analysis.

### Passive microrheology by particle tracking

Particle tracking was conducted according to Chew et al. and Hart et al.^26,49^ in order to infer information regarding the viscoelastic properties of the biofilms at the microscopic scale. When applied to biofilms, the principle of the method is that the extent to which the cells (or alternatively sphere probes introduced in the biofilm) are displacing or oscillating under static conditions (i.e. no flow) is dependent on their inclusion in the biofilm 3D structure. Cells tightly attached to the substrate and included in the matrix present lower displacement than cells not adhered or detached. The movement of the cells under static conditions is only resulting from their Brownian motion and can be quantified by particle tracking (i.e. video microscopy). Further processing of the trajectories is necessary to extract quantitative and detailed information about cell displacement.

In this study, all videos were acquired at 25 °C under static conditions (i.e. no flow in the system) at day 2, 4, 7 and 11 in transmission mode using a 20× magnification lens (see confocal section) and an AxioCam MRm (Carl Zeiss microscopy GmbH, Jena, Germany). Each image (512 × 512 pixels; 164 × 164 µm; 8-bit) was acquired every 0.042 s (camera exposure time = 2 ms) with a spatial resolution of 0.32 µm. The total length of the videos was typically 2–3 min. Only the cell layer directly in contact with the substrate was imaged (from 0 to ~ 15 µm above the support).

Videos were further imported and pre-processed in FIJI and particle tracking was performed using the plugin Trackmate^50^. The grey scale was first inverted, a local contrast enhancement was then applied and finally the background was removed using the function “rolling ball radius” equal to 10 (pixels). Once imported in Trackmate, a blob radius of 4–7.5 µm, and the Log (plain Laplacian of Gaussian filter) detector were used to detect the particles. The “simple LAP (Linear Assignment Problem) tracker” method and a linking maximal gap-closing distance of 1 µm were selected for all tracks. Once the tracks were reconstructed, further filters were used to exclude tracks of low quality (e.g. erroneous spot detection and short tracks). Only spots with a quality higher than 1.5 and the tracks presenting a number of point higher than 1000 (≥ 40 s track length) were selected for the analysis. The displacement of each detected cell was calculated (in µm) as the distance between the last and the first position of the track in time^50^. After the applications of the filters, a number of cell tracks comprised typically between 50 and 300 was obtained for each video. Three videos were acquired in random positions of each channel in a flow-cell (e.g. inlet, middle and outlet). The displacement of the cells in the biofilms at the different shears was visualized by pooling the log-transformed data at each condition and using the Kernel density plot^51^ in order to estimate the probability density function of cell population.

### Cohesiveness—Erosion test

The cohesion of the biofilms cultivated at different hydrodynamic regimes was tested using a protocol similar to the one described in Paul et al.^16^. After seven days of growth, the biofilms were subjected to an increasing set of shears (9, 18, 36 and 71 mPa) with each step lasting 10 min. The range of shear stress covers the maximal operative range of the peristaltic pump used in this system and was lower with respect to the one used in Paul et al.^16^. After each step, the biofilms were scanned and the biomass lost due to detachment was quantified with respect to the initial biomass (pre-test) present in each channel has follow:

3 $${Detached}_{Biovolume(x)}= \frac{{Biovolume}_{i}-{Biovolume}_{shear(x)}}{{Biovolume}_{i}}\cdot 100$$

where *Biovolume*_*i*_ represents the biovolume measured at day 7 before the erosion test begun and *Biovolume*_*shear(x)*_ is the biovolume measured after each step of the erosion test with *x* ranging from 9 to 71 mPa.

The vertical profile of cell coverage was also calculated in order to spatially localize the loss of cell biomass at each shear step.

### Statistics

Statistics was performed using GraphPad prism 5.0 (San Diego, CA, USA) and the R software^52^. Each experiment was conducted on at least three independent biofilms and data are reported as mean and standard deviation. To evaluate the significance of mean differences among shear conditions, One-way and Two-way ANOVA followed by the Tukey’s post-hoc test for multiple comparisons were conducted. Conditions sharing the same letter combinations are not statistically different whereas conditions presenting no letter in common are statistically different. The level of significance was always set at 5%.

## Supplementary Information

Supplementary Information.

## Data Availability

All data generated or analysed during this study are included in this published article (and its Supplementary Information files).
