# Supplementary material for: Shear stress affects the architecture and cohesion of Chlorella vulgaris biofilms
Source: Sci Rep. 2021 Feb 17;11:4002. doi: 10.1038/s41598-021-83523-3 (PMC7889892; doi:10.1038/s41598-021-83523-3)
Supplement: Supplementary file 1 — Supplementary Information. [file 41598_2021_83523_MOESM1_ESM.docx]

**Supplementary information for the article:**

**Shear stress affects the architecture and cohesion of *Chlorella vulgaris* biofilms**

Fanesi A.^1^, Lavayssière M.^1^, Breton C.^1^, Bernard O.^2^, Briandet R.^3^, and Lopes F^1,*^.

^1^Laboratoire Génie des Procédés et Matériaux (LGPM), CentraleSupélec, Université Paris-Saclay, 91190 Gif-sur-Yvette, France

^2^Biocore, INRIA, Université Côte d'Azur, 06902 Sophia Antipolis Cedex, France

^3^Micalis Institute, AgroParisTech, INRAE, Université Paris-Saclay, 78350 Jouy-en-Josas, France

*****Correspondence: [filipa.lopes@centralesupelec.fr](mailto:filipa.lopes@centralesupelec.fr); Tel.: +33175316112

**
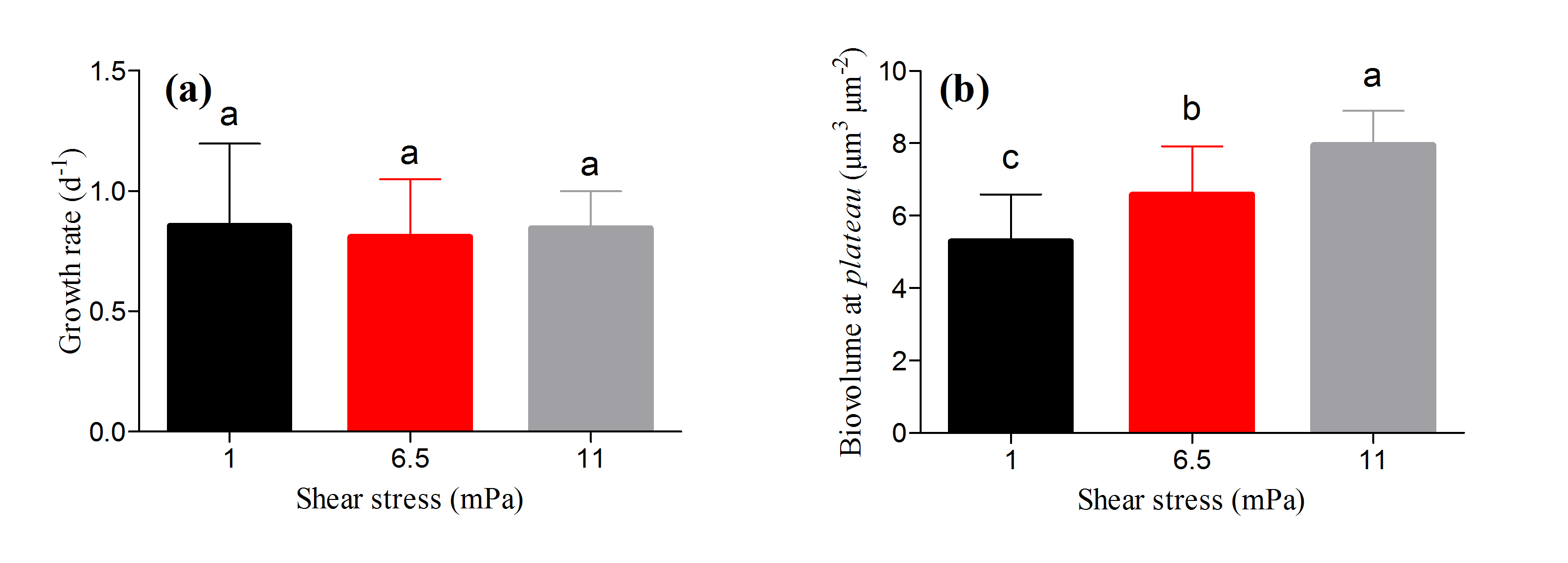
Figure S1.** Growth parameters of *C. vulgaris* biofilm grown at different hydrodynamic shears: 1.0, 6.5 and 11.0 mPa. Panel (a) depicts the growth rate of the biofilms and panel (b) the biovolume of biofilms at the *plateau* of the curves. The two growth parameters were determined by fitting the logistic functions to the biovolume vs. time curves. The results are reported as the mean and standard deviation of at least three independent biological replicates. Bars with different letters represent statistically different means (*p*<0.05) as determined by pair-wise comparisons after two-way ANOVA.

**
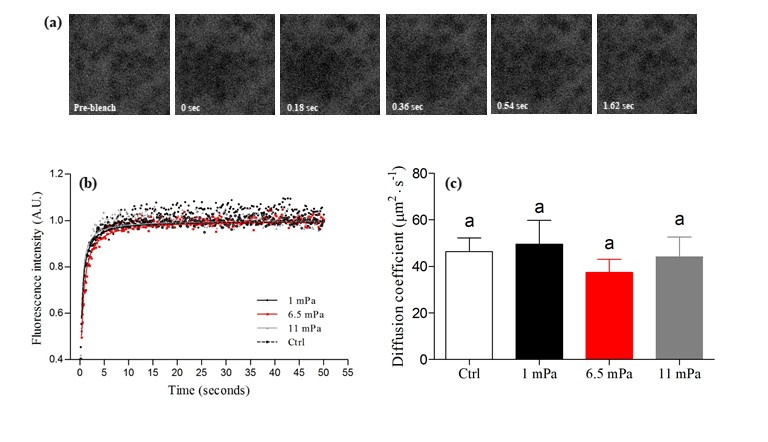
**

**Figure S2.** Example of FRAP measurements. In (a) representative image sequences starting just before the bleaching pulse and showing the evolution of fluorescence recovery are reported. In panel (b), the intensity of fluorescence and the respective fitting functions are plotted against time for the three different shear conditions. In panel (c) the diffusion coefficients in the biofilms and in growth medium (ctrl) are reported (n≥5). FRAP curves were obtained using a commercial CLSM using a 70kDa FITC-Dextran as a probe molecule. The FITC-Dextran was bleached until the fluorescence signal reached 40% of the initial intensity and the recovery was followed over 50 seconds. The curves were subjected to a double normalization and finally fitted with the Soumpasis model and the diffusion coefficient calculated as *D = r^2^/(4·τ_d_)*, with *r* being the radius of the bleached spot and *τ_d_* the diffusion time. For all measurements, the diffusion coefficient was obtained at the deepest layers of the biofilms (the one closer to the substratum).


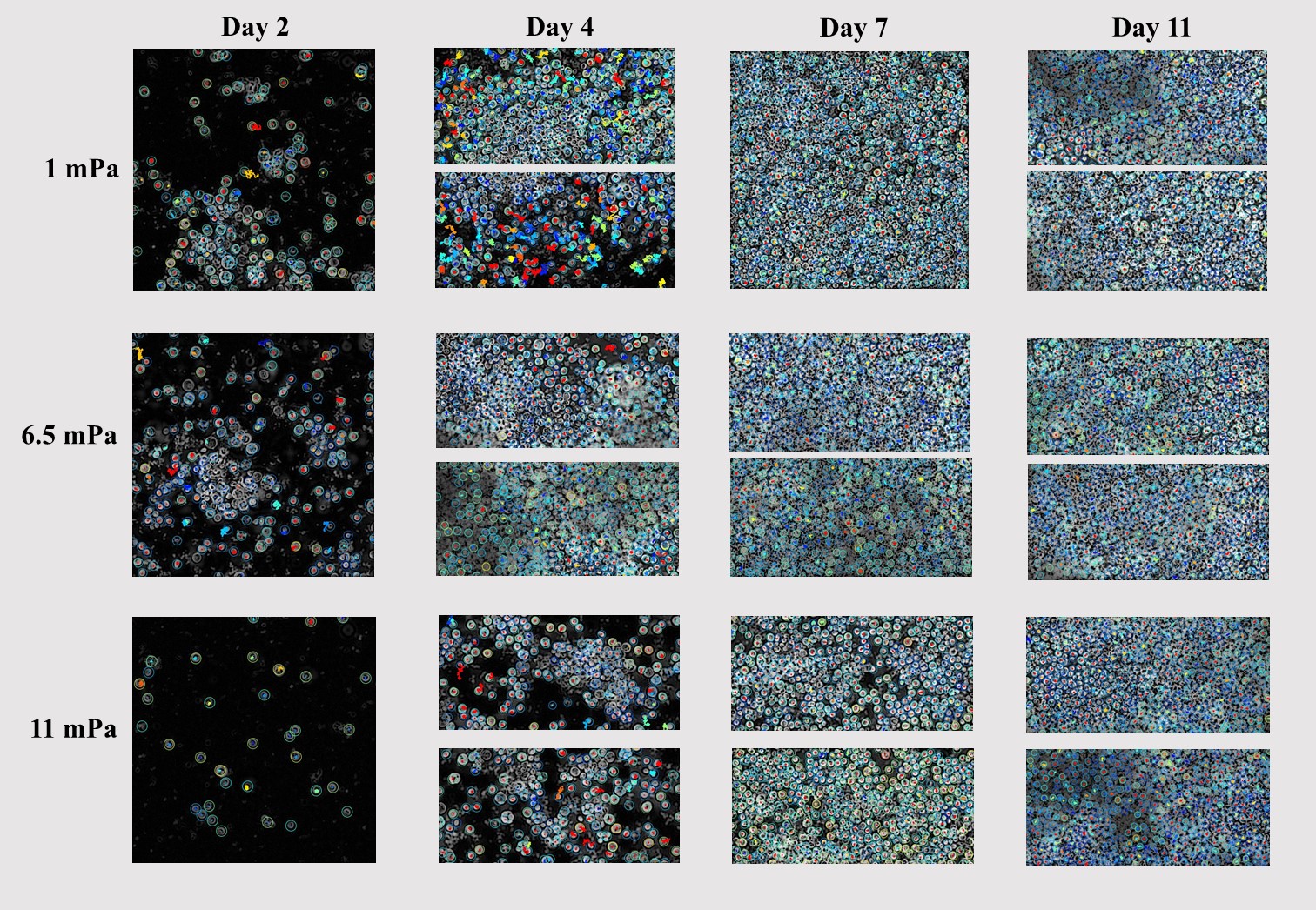


**Figure S3.** Representative cell trajectories obtained using the particle tracking method. The spots (i.e. the cells) detected and the trajectories are reported for day 2, 4, 7 and 11 for the biofilms developed at three hydrodynamic regimes (1.0, 6.5 and 11.0 mPa). Only trajectories that lasted for at least 40 s were considered for the final analysis. Images were cropped to speed up the analysis if the number of particles to be tracked was too high. The feature displacement was calculated from cell trajectories as the difference between the last and the first point of the trajectory.


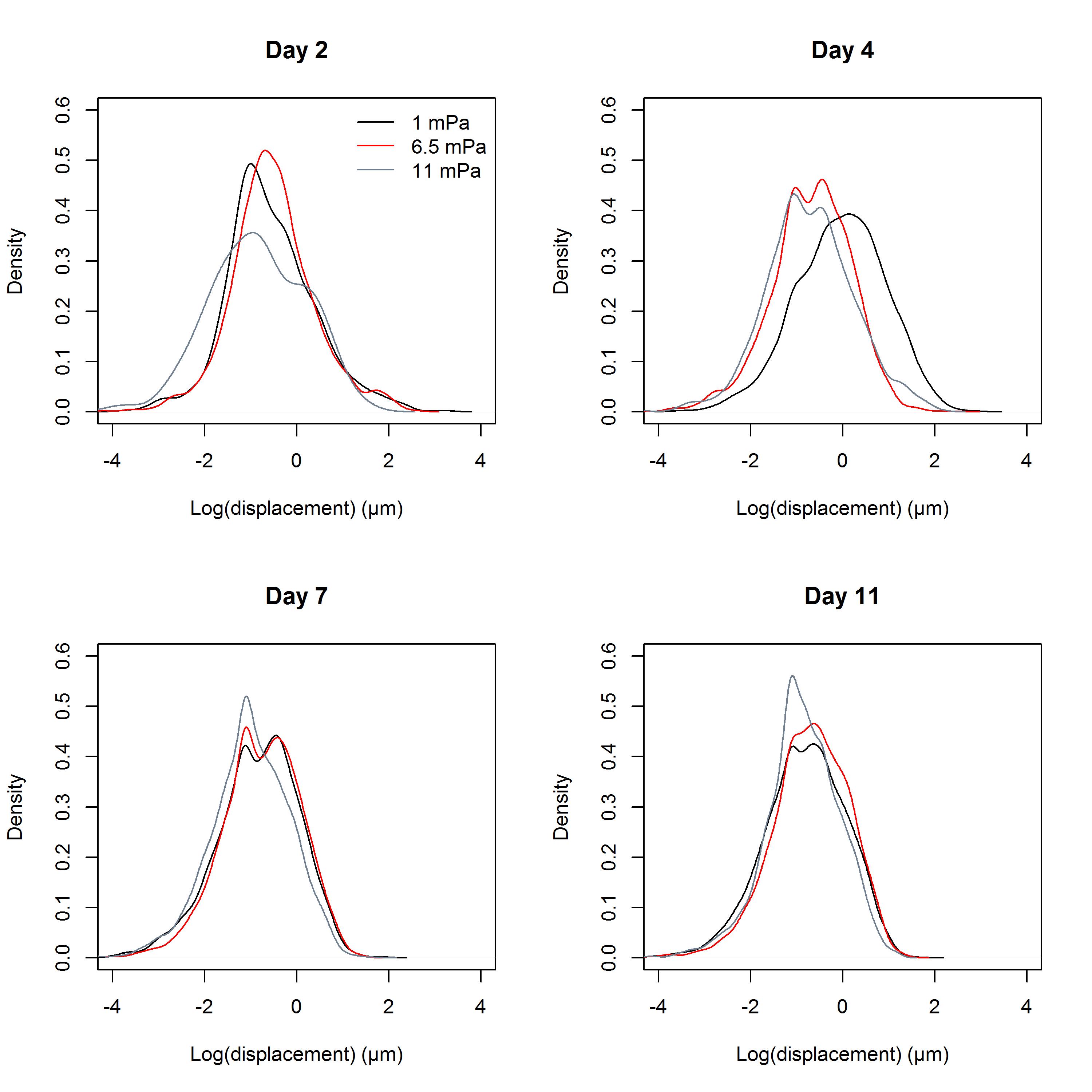


**Figure S4.** Kernel density estimation of displacement data. The displacement was calculated from cell trajectories obtained from particle tracking as the difference between the last point and the initial point of the trajectory. Data were further processed with a log transformation. The smoothing kernel used was the Gaussian one and the smoothing bandwidth was selected using the default parameter in the R function.
